# GAMEC – a new intensive protocol for untreated poor prognosis and relapsed or refractory germ cell tumours

**DOI:** 10.1038/sj.bjc.6604041

**Published:** 2007-10-16

**Authors:** J Shamash, T Powles, W Ansell, D Berney, J Stebbing, K Mutsvangwa, P Wilson, S Asterling, S Liu, P Wyatt, S P Joel, R T D Oliver

**Correction to**: *British Journal of Cancer* (2007) **97**, 308–314. doi: 10.1038/6603865

Owing to an author&apos;s error, the name of the fourth author of the above paper, Dan Berney, was omitted when originally published in Volume 97, Number 3. We are now happy to correct this mistake; the full, correct authorship is now listed above.

